# The Current Status and Factors Associated With Implanon Service Provision by the Health Extension Workers at the Health Post Level, Wolaita Zone, Southern Ethiopia: A Cross-Sectional Study 

**Published:** 2017-03

**Authors:** Ketsela Desalegn, Eskindir Loha, Mengistu Meskele

**Affiliations:** 1 Path Finder International, Southern Region, Hawassa, Ethiopia; 2 School of Public and Environmental Health, Hawassa University, Hawassa, Ethiopia; 3 School of Public Health, College of Health Sciences and Medicine, Wolaita Sodo University, Wolaita Sodo, Ethiopia

**Keywords:** Implanon, Service Provision, Family Planning, Ethiopia

## Abstract

**Objective:** Family Planning is often taken as one of the “Magic Bullet” interventions owing to its high impact and wide reaching nature in achieving multiple goals. This study aimed to assess the current status and the factors associated with health post level Implanon service provision through trained health extension workers in Wolaita zone, southern Ethiopia.

**Materials and methods:** A cross sectional study was conducted among trained health extension workers in Wolaita zone in February 2013. A simple random sampling technique was used to identify a total of 285 trained HEWs. First bivariate, then multivariate logistic regression model along with 95% confidence interval was used to see the independent effect of factors associated with current Implanon service provision by the health extension workers.

**Results:** Currently, the number of Implaon providing trained health extension workers in Wolaita was 264(45.8%). Distance of health post from district health offices and health center, turnover of trained health extension workers in the health post, interest of trained health extension workers in providing Implanon and their job satisfaction to serve as a health extension workers and availability of service delivery guidelines and teaching aids were associated with the current provision of Implanon by health extension workers.

**Conclusion:** Implanon provision among trained health extension workers was affected by different factors. Hence, improving the working conditions of trained health extension workers, regular and periodic facilitative supervision, availing service delivery guidelines and improvement of health management information system are recommended.

## Introduction

Universal access to family planning, sexual and reproductive health-care got much emphasis in sustainable development goals (SDGs). Moreover, family planning is a good tool to achieve the target set at SDG by 2030, to reduce the global maternal mortality ratio to less than 70 per 100,000live births (1).

Ethiopia is still made little progress towards alleviating maternal mortality rate (MMR) in the millennium development goals (MDG) level, though a concerted effort to achieve most of the national and international developmental targets. The MMR was 676/100,000 live births, a level not adequately fulfilled the required level for the 2015 MDG target (2). Currently, according to Maternal and Perinatal Health Profile in the year 2013 the MMR in the country was 420/100,000 live births (3). Fortunately, among other measures, the vast majority of maternal and newborn deaths can be prevented with proven interventions to ensure that every pregnancy is wanted using modern contraceptives provision (4- 6). 

To this end, the Federal Minister of Health (FMOH) has lately adopted the task- shifting strategy in which the long acting family planning (FP) service provision is delegated to the Health Extension Workers (HEWs) at the health post (HP) level (6- 8). Moreover, the FMOH has taken bold decisions to initiate Long Acting Reversible Contraceptive (LARC), the Implanon. This service provision was transferred from health professionals in health centers and hospitals to Implanon trained HEWs in the HP (6, 9). 

Wolaita is one of the few zones in which the first round Implanon scale-up at the health post level for HEWs was conducted in Ethiopia. Currently majority of HPs of all the districts of Wolaita have been assumed to be staffed with trained HEWs on Implanon insertion. Hence, the program will be expected to be running in all the rural districts of the zone at the HP level through trained HEWs. This resource intensive initiative has been supported with resources from the public sector & other partners. However, the required support was characterized by considerable irregularity (i.e. dispensing logistics for the services, supervisory support by trained supervisors, readily availing removal services on demand for the needy etc) since the start of the program ( 9 and 10).

It is believed that giving women access to Implanon as a long-term method at HP level will help further improve the contraceptive prevalence rate and reduce unmet needs, which are both MDG indicators of maternal health (6, 7). Though, ensuring the continuity of the HEWs driven Implanon provision at the Health Post level through sustained technical and logistical support is of paramount importance (5, 8). However, current Implanon provision through the HEWs was not fully documented, and factors affecting it were not studied to suggest better options of its activity.

## Materials and methods

***Study area and setting:*** A facility based cross- sectional study design was used. The study was conducted on February 2013 in wolaita zone, one of Southern Nations, Nationalities and Peoples Region (SNNPR) of Ethiopia, and known for its high population density. Based on the most recent census (2007), which was conducted by the Central Statistical Agency of Ethiopia, the projected total population of the zone was 1,750, 079 with 863,043 males and 887,036 females on July, 2012 (11). According to the Wolaita Zone health Department report, there were 332 Health posts staffed with 742 Health workers and 681 HEWs in 324 HPs destined to serve its needy population in the 12 rural districts alone (excluding the urban ones). Out of the 681 total HEWs, 637 (93.5%) of them were trained on Implanon insertion techniques as of the start of the program. 

***Sampling*** *:* In determining the sample size (n) for such a small size population of trained HEWs (n of 637) for the study, we used a single population proportion along with a finite population correction formula. To this end, the proportion of Implanon service provision by the Health Extension Workers at the Health Post level was assumed to be 50% because there was no study in the local context in the issue under the caption. Therefore, N (population size) of 637, Z (confidence level) of 1.96, E (random error) of 4.5% .The calculated sample size was 272 and adding 5% non-response, the required minimum sample size was 285. 

The target population for this survey were trained HEWs who have been supported by the FMOH and partners to start delivering Implanon at the HP level in the 12 district (n= 637). The study subjects were the randomly selected trained HEWs drawn from the six district of the zone. The sampled population (n) was further allocated to each of the 6 district proportional to the size of the district .For this proportional allocation a sampling fraction (n/N) was used for all the 6 district, making it proportional to the size of each. We used a sampling frame for the final step on which the study units (trained HEWs) were listed under the six rural districts. Subsequently the eligible HEWs were selected on the basis of simple random sampling technique. 

***Operational Definitions***

*Skill based Implanon training:* A six days competency based training for health extension workers that include skills in client counseling, basics of infection prevention, client screening for legibility and technical competence to be exercised on the arm models, before practicing on actual client under strict coaching by experienced staff. 

*Current Implanon service provision:* Provision of Implanon insertion services during the last 90 days (3 months) in a health post by a trained health extension workers.

***Data collection and instrument: ***Data were collected through structured interviewer administer questionnaires. Then it was translated in to the local language of the region, the Amharic, by other expert and back translated in to English to check consistency of the language. Pre testing of the questionnaire was conducted in “Sodo zuriya” district which is similar setting with the study area. Moreover, pertinent modifications were made following the pre-testing exercise. Six data collectors, who were fluent in local language had diploma nursing were identified, hired and trained for two days. Through the guideline produced for this purpose, they were also introduced to the overall objective of the study, the instruments & the procedures to be utilized during the survey. The six data collectors were assisted and supervised by two supervisors with first degree level of training in public health. Supervisors were also in charge of ensuring that data were correctly filled, checked, corrected and compiled on a daily basis. 

***Data*** ***analysis and interpretation:*** The data were checked and edited ,then coded , entered in to SPSS, cleaned and checked for outliers and completeness, after which were analyzed using SPSS version 17 (SPSS Chicago, IL.USA). Descriptive statistics for each variable was undertaken to show frequencies and percentages. Bivariate logistic regression model along with 95% confidence interval and odds ratio were employed to examine the association between the outcome and each explanatory variable. Further, to assess the relative net effect of each predictor variable on the outcome variable controlling the others, the multivariate logistic regression model has been employed. 

The independent variables which had a p-value less than 0.1 in the bivariate logistic analyses were included in the multivariate analysis later on. Furthermore, the multicollinearity effect among the included variables in each model fitting was tested and the variance inflation factors (VIF) were found to be far less than the cut off value (of ten). 

***Ethical consideration*** ***:*** Before the study was started, ethical clearance was obtained from the board of review committee at Hawassa University, school of public and environmental health. A permission letter to conduct the study was obtained from Wolaita zone health department and the 6 district health offices and presented to all participants and stake holders by the data collectors and principal investigators as needed to secure their willingness. In the letter the purpose of the study and its procedures were fully explained to the officials at all levels. Likewise, HEWs who were selected for the interview were informed about the purposes and the procedures of the intended survey, and they were enrolled in the study only if they provide their verbal consent.

## Results

***Socio-Demographic Characte*** ***ristics: ***Of thetotal 285 trained HEWs identified as eligible to participate, 264 (92.6%) gave consent and participated in the study. According to the survey, age of the study subjects ranged from 18 – 40 years with a mean age of 25.2 **±** 3.5 years, 215 (81.4%) of them educated 10 + 1 and 189(71.6%) were married. 

Two hundred twenty one (83.7%)) were recruited from the same kebele where they were serving at the time of data collection, while 26(9.8%) from nearby kebele and 16 (6.1%) were from nearby district towns. All the respondents had more than two years of work experience. One hundred fifteen (43.6%) of them served for two to five years and 149(56.4%) were served for more than five years. More than half 141 (53.4%) of the HEWs declared that the health posts, where they were working at the time of the survey were over 10kms away from their respective district health offices and 136 ( 51.5%) of the study participants were working at the health posts distant 5kms and above from the nearby health centers. 

***HEWs training, current Implanon provision and attitude towards Implanon provision*** ***:*** Only 37 (14.0%) of the study participant HEWs had got Implanon insertion training during their regular (pre-service) training program. On the other hand around 256 (97%) of the respondents’ colleagues had got an in-service training on Implanon insertion service delivery. Concerning the turnover of trained HEWs from the HPs, though 151 (59.0%) of the participants were working together with their Implanon trained colleagues, the remaining 105 (41.0%) responded that at least one of them had currently left out of the same HP at the time of the survey.

Moreover, although 148 (56.1%) of the trained HEWs explained the in-service training on Implanon insertion they were provided was inadequate, 260 (98.5%) of them had ever delivered Implanon insertion service sometime after their training. However, of all the 264 study participants only 121(45.8%) of them were currently (for the last 90 days prior to the date of the data collection) providing the Implanon insertion service in their respective HPs, and 143 (54.2%) of the trained HEWs did not ever provide the service during the same period. This is suggestive of the progressive decline of the Implanon insertion service at the HP level through trained HEWs in time (Table 1).

***Consumables and Logistics Supplies for Implanon Provision at HP Level:*** Out of the total 264 HEWs of the six selected district of Wolaita zone, 158 (59.8%) had stokes of Implanon in their HP. Twenty five (15.8%) of those who had stokes of Implanon reported that their stokes has expired. Furthermore, 171 (64.8%) of all the study participants also explained that they had stokes of gloves for Implanon provision. However 123(46.6%) of the trained HEWs complained that they had no stokes of bottles of Iodine tincture to provide Implanon insertion, and 132 (50%) of the study subjects had no stokes of vials of Lidocaine for the provision of Implanon insertion service. 65.5% and 74.2% of the HEWs had stokes of pieces of syringes and first aid plasters, while 33.7% and 25.8% had no stokes of syringes & plasters required respectively for Implanon provision. 

District health office (32.6%), partners operating in the district (20.5%), district health office and partners jointly (33.3%) were stated by the HEWs as the common suppliers of the contraceptives, consumables and logistics so far for the provision of Implanon insertion services at HP level.

**Table 1:** Respondents’ training, current Implanon provision and attitude through trained HEWs

**Characteristics (n = 256)**	**Frequency (n = 264)**	**Percent**
The other colleague also had an in-service training on Implanon	256	97
Both trained HEWs working in the same HP now (n = 256)		
Yes, both we are in the same HP	151	62
No, she has left the HP now	62	24.2
No, I have left the HP now	16	6.3
No, both we have left the HP now	27	10.3
The in-service Implanon training was adequate	116	43.9
Yes	116	43.9
No	148	56.1
Provided Implanon insertion at your HP after the training		
Yes	260	98.5
No	4	1.5
Currently providing Implanon insertion services at your HP		
Yes	121	45.8
No	143	54.2
The last time you provided Implanon insertion service at your HP		
< 90 days	121	45.8
90 days - 6 months	63	23.9
> 6 months	74	28
Do not remember	6	2.3
You are interested to provide Implanon at your HP		
Yes	112	42.4
No	128	48.5
Neither interested nor disinterested	24	9.1
Satisfied by your profession as a HEW		
Yes	112	42.4
No	123	46.6
Neither interested nor disinterested	29	11

**Table 2:** Consumables and Logistics Supplies for Implanon at the HP Level

**Consumables and Logistics Supplies**	**Frequency (n = 264)**	**Percent**
Health post currently has stokes of Implanon	158	59.8
The status of the expiry date of Implanon available in your HP (n = 158)		
Expired	25	15.8
Not expired	133	84.2
Health post currently has stokes of Gloves for Implanon provision	171	64.8
HP currently has bottles of Iodine tincture for Implanon provision		
Yes	138	52.3
No	123	46.6
Do not know	3	1.1
HP currently have vials of Lidocaine for Implanon provision		
Yes	128	48.5
No	132	50.0
Do not know	4	1.5
HP currently has pieces of Syringes for Implanon provision		
Yes	173	65.5
No	89	33.7
Do not know	2	0.8
HP currently has pieces of first aid plasters	196	74.2
Provider/s of the stated logistic supplies so far		
District health office(FMOH)	86	32.6
Partner operating in the district	54	20.5
District health office and partner	88	33.3
Community	36	13.6
The last time you were provided with logistic supplies		
< 1 year	100	37.9
> 1 year	161	61.0
Do not remember	3	1.1

However, the last time the participants were supplied with the stated logistics supplies for the provision of Implanon service was more than one year for the majority (61.0%) of the participants. This indicates that the provision of consumables and logistics supplies by the responsible parties lacks sustainability for the continuation of Implanon insertion delivery among the community in the study area at HP level (Table 2). 

***Workload, Support and Supervision for Implanon Provision at HP level*** ***:*** Among all the HEWs surveyed, 245 (92.8%) were assisted by other staff members on the service provision in their HPs. The majority (62.9%) of these respondents explained that the assistance for Implanon insertion service provision is obtained from the other HEW of the same HP. Furthermore, 35.9% of the study participants were assisted by health development agents (HDAs) in addition to a colleague HEW in the same HP. Regarding the work load situation at the HP level, it was explained as too much work by 153 (58.0%) of the respondents, although 41.3% of their counterparts described it as a manageable amount of work. The majority (59.5%) of the whole participants made it clear that they haven’t received a regular facilitative supervisory visit for Implanon service delivery so far. Moreover, out of those who were supervised so far, only 27(25.2%) of them had received a regular facilitative supervisory visit during the last three months (Table 3).

***Service delivery guidelines and recording of Implanon provision through HEWs*** ***:*** More than half, 138 (52.3%) of the trained HEWs had no Implanon insertion service delivery guideline for HEWs in Ethiopia in their respective HPs. Of those who had the guideline in hand, half (51.6%) of them did not use the guideline as a reference during the service provision. 

**Table 3:** Workload, Support and Supervision for HP level Implanon provision

**Characteristics (n = 264)**	**Frequency**	**Percent**
You had other staff members assisting you in your HP		
Yes	245	92.8
No	19	7.2
Who assisted you in the provision of Implanon service (n = 245)		
Only one additional HEW	154	62.9
Volunteers only	2	0.8
One additional HEW & health development agents (army)	88	35.9
Others	1	0.4
Describe your work load		
Too much work	153	58.0
Manageable amount of work	109	41.3
Do not know	2	0.8
Received a regular facilitative supervisory visit about Implanon sofar		
Yes	104	39.4
No	157	59.5
Do not remember	3	1.1
Received supervisory visit in the past 3 months for Implanon (n = 107)		
Yes	27	25.2
No	77	72.0
Do not remember	3	2.8
Technical supervisors to HP come from (n = 104)		
Nearby Health center	50	48.1
District health office	21	20.2
Zone health department	8	7.7
Regional health bureau	4	3.8
Partner organization	21	20.2
Clients got removal services at the HP level by a provider from the HC	62	23.5
The last time a provider came to deliver removal services to the HP (n = 62)		
< a month ago	7	11.3
Between one and three months	30	48.4
> three months	23	37.1
Do not remember	2	3.2
Supervisor is available to help you whenever you need his/her assistance		
Yes	92	34.8
No	167	63.3
Do not know	5	1.9

For the question whether they had Implanon medical eligibility criteria checklist at their respective HPs or not, far more than half (65.9%) of the participants responded the absence of the check list. Besides this 75.6% of those who had the document never used as a reference in their career. Most (64.8%) of the trained HEWs had desktop reference materials for clients’ education about FP methods, though, 40.9% of which did not refer it when providing FP service delivery (Table 4).

***Determinants of Implanon Provision by the HEWs*** ***at Wolaita zone:*** Respondents who were working in the HPs at a distance of less than 10kms from their respective district health offices were found to be 16.8 times more likely to provide Implanon insertion service currently as compared to those who are working in HPs situated over 10kms from the district health offices (AOR = 16.82; 95% CI: 4.90-57.80). Moreover, the HEWs who are working in the health posts found at a distance of less than 5 kms were 5.21 times more likely to provide Implanon insertion service currently as compared with those who are working in the HPs at a distance of more than 5kms from the nearby HCs (AOR = 5.21; 95% CI: 1.55- 17.49).

**Table 4:** HP level Implanon provision through HEWs in relation to service delivery guidelines, recording and monitoring

**Service delivery guidelines, recording and monitoring(n = 264)**	**Frequency**	**Percent**
Have the Implanon insertion service delivery guideline for HEWs, Ethiopia	126	47.7
Use the guideline document as a reference (n = 126)	61	48.4
Implanon medical eligibility criteria checklist available at your HP	82	31.1
Use the checklist as a reference (n = 90)	22	24.4
Have desk top reference for clients' education on FP methods	171	64.8
Use the desk top reference materials as a reference (n = 171)	101	59.1
Have FP service registration books at your HP	258	97.7
Total number of Implanon clients registered for last six months (n = 184)		
< 10	38	20.7
Between 11 and 20	41	22.3
Between 21 and 30	58	31.5
> 30	47	25.5
Implanon performance report incorporated in last quarter report to the district	29	11.0
Reason for not incorporating the Implanon in the final quarter report		
There were no mothers served on Implanon during the quarter	29	12.3
The report format lacks questions about Implanon served mothers	161	68.5
The district office did not request us to include report about Implanon	45	19.1

As well, each of the pre-service (regular) training inclusion of Implanon insertion and adequacy of the in-service training had significant association with the current provision of Implanon as explained by the (Crude OR (COR = 3.82; 95% CI: 1.77- 8.27) and COR = 6.74; 95%CI: 3.92-11.59) respectively), their net effect remained statistically insignificant when all the other variables were controlled (Table 5).

**Table 5:** Background characteristics, Training and Attitude Related Determinants of Implanon Service Provision through HEWs

Variables (n = 264)	Current Implanon Provision		Crude OR (95% CI)	Adjusted OR (95% CI)
	Yes	No		
**Service year as a HEW**				
** 2 – 5years**	**57**	**58**	**1.31(0.80- 2.13)**	**1.58(0.53-4.67)**
** >5 years **	**64**	**85**	**1.0**	**1.0**
**Distance of HP from district office**				
** < 10 km**	**109**	**14**	**83.70(37.15-188.54)** ***	**16.82(4.90-57.80)** ***
** > 10 km**	**12**	**129**	**1.0**	**1.0**
**Distance of HP from the nearby HC**				
** < 5 km**	**103**	**25**	**27.0(13.95-52.31)** ***	**5.21(1.55-17.49)** **
** > 5 km**	**18**	**118**	**1.0**	**1.0**
**Implanon included in Pre-service **				
** Yes**	**27**	**10**	**3.82(1.77-8.27)** **	**3.23(0.43-24.23)**
** No**	**94**	**133**	**1.0**	**1.0**
**Both HEWs working in the same HP **				
** Yes, both of us working **	**91**	**60**	**1.0**	**1.0**
** No, at least one of us has left ** @	**30**	**75**	**0.26(0.16-0.45)** ***	**0.16(0.05-0.48)** **
**In-service Implanon training adequate**				
** Yes**	**82**	**34**	**6.74(3.92-11.59)** ***	**2.76(0.95-8.07)**
** No **	**39**	**109**	**1.0**	**1.0**
**Interested in providing Implanon at HP**				
** Yes **	**90**	**22**	**1.0**	**1.0**
** No**	**19**	**109**	**0.04(0.02-0.08)** ***	**0.13(0.04-0.41)** **
** Neither interested nor uninterested**	**12**	**12**	**0.24(0.10-0.62)** **	**0.37(0.07-1.86)**
**Satisfied by the profession as a HEW**				
** Yes **	**80**	**32**	**1.0**	**1.0**
** No**	**28**	**95**	**0.12(0.07-0.21)** ***	**0.12(0.04-0.40)** **
** Neither satisfied nor dissatisfied**	**13**	**16**	**0.33(0.14-0.75)** **	**0.28(0.05-1.41)**

*P < 0.05;

*** P < 0.001;

** P < 0.01;

@ = I left the HP, My colleague (she) left the HP, and both of us left the HP.

**Table 6:** Support, Work load and Supervision Related Variables Associated with Implanon Provision through the HEWs

**Variables (n = 264)**	**Current Implanon ** **Provision**		**Crude OR** **(95% CI)**	**Adjusted OR** **(95% CI)**
	**Yes**	**No**		
Other staff members assist you in the HP				
Yes	119	126	1.0	1.0
No	2	17	0.13(0.03-0.55)**	0.16(0.02- 1.15)
Describe your work load				
Too much work	44	109	1.0	1.0
Manageable amount of work	76	33	5.71(3.33-9.77)***	5.67(2.84- 11.32)***
Do not know	1	1	2.48(0.15-40.49)	11.41(0.33-395.71)
Receive supervisory visit on Implanon				
Yes	75	29	6.41(3.70-11.09)***	2.48(1.08-5.69)*
No	46	114	1.0	1.0
Feel a supervisor is available to support you whenever you need his/her assistance				
Yes	64	28	1.0	1.0
No	55	112	0.22(0.12-0.37)***	0.70(0.30-1.64)
Do not know	2	3	0.29(0.05-1.84)	0.46(0.05-4.59)
HEWs have the Implanon insertion service delivery guideline for HEWs in Ethiopia				
Yes	83	43	5.08(3.01-8.58)***	2.28(1.15-4.53)*
No	38	100	1.0	1.0
Desk top reference materials available for clients' education on FP methods				
Yes	105	66	1.0	1.0
No	16	77	0.13(0.07-0.24)***	0.18(0.09-0.39)***
You incorporated your implanon performance in your recent quarter report to the district				
Yes	27	2	20.25(4.70-87.18)***	11.92(2.04- 69.52)**
No	94	141	1.0	1.0

*** P < 0.001;

** P < 0.01,

* P < 0.05

***Support, Work load and Supervision Related Determinants of Implanon provision through the HEWs*** ***: ***The trained HEWs who explained their work load as manageable were 5.67 times more likely to provide the Implanon insertion service as compared with those who described it as too much work (AOR = 5.67;95%CI: 2.84-11.32). Besides, the HEWs who had received a regular facilitative supervisory visit about Implanon service were 2.48 times more likely to provide Implanon insertion service than those who had not received (AOR = 2.48; 95% CI: 1.08-5.69). 

With regard to the guidelines and reference materials about FP, the respondents who had the Implanon insertion service delivery guideline for HEWs in Ethiopia were found to be 2.28 times more likely to provide Implanon insertion service as compared with those who did not have the guideline (AOR = 2.28; 95% CI: 1.15-4.53). Besides, those trained HEWs who had incorporated their Implanon performance in their last quarter report to their district were 11.92 times more likely to be currently providing Implanon insertion services (AOR = 11.92; 95% CI: 2.04- 69.52) (Table 6).

## Discussion

Though all the participants of this study were those HEWs trained on Implanon insertion, just below half (45.8%) of them were found providing Implanon insertion service during the time of the data collection. This result was also observed to be far less than the service provision performance that was observed immediately after they had had the training (98.5%). The study also showed that 51.9% of the trained HEWs provided the service earlier than three months before the study time. These all indicated the progressive decline of the Implanon insertion service at health post (HP) level through the trained HEWs. Besides considerable number of the trained HEWs (46.6%) of this survey were found to be dissatisfied to be working as a HEW and (48.5%) of them had lost any interest to provide Implanon insertion service. This result was found to be below the finding of another study conducted in Ethiopia on “assessment of the extent and determinants of functionality of HEWs in East Gojjam Zone, Amhara regional state”; that 67.6% of the HEWs had no satisfaction as a HEW (11).

One hundred six (40.2%) of the respondents had no stokes of Implanon in their respective HPs and of those 158 (59.8%) who had stoke of Implanon, 25(15.8%) of them had an expired one. Ninety three (35.2%) of the respondents had no stokes of gloves and 123(46.6%) had also no bottles of Iodine tincture for Implanon provision. This outcome was supported by the finding of the study done on the working condition of HEWs in Ethiopia that some HPs have no supplies at all and in a number of HPs in Oromia, Amhara etc. where there are supplies, some major items/drugs such as contraceptives have been missing (12). Similarly a study in Bahir-Dar special zone showed unavailability and shortage of different contraceptive methods in the service delivery posts (13). 

Most 154(62.9%) of the trained HEWs were assisted by only one additional HEW in the HP for Implanon insertion service provision. While in East Gojjam, 57% had claimed that they were staffed with two HEWs and one guard (11). Regarding the work load, 153(58.0%) of the participant HEWs reported as having to deal with “too much work”at their HP, while 109(41.3%) of them declared it as “manageable amount” of work. The reported “work overload” that partly emanates from other emerging government priorities of the HEWs was shown in this study to negatively affect the dependent factor.

Besides, the majority (59.5%) of the trained HEWs haven’t received facilitative supervisory visits on Implanon service delivery so far. Of those who were supervised, only 27(25.2%) of them had received a regular facilitative supervisory visit during the last three months. Inadequate supervision on FP services from higher institutions was also documented in Jima and Bahir-Dar; where all health service delivery points in Jimma and except two non-governmental and one Governmental clinic in Bahir-Dar the remaining had not received supervisory visit within three months prior to data collection time (13). In contrast to this, the study in East Gojjam disclosed that, of those 84.5% HEWs who had been supervised, 58.8% were supervised at a frequency of every month (11).

Among those who had received supervisory visit, 50(48.1%) of them were supervised by nearby health center, 21(20.2%) by partner organizations and the district health offices, and the rest 12(11.5%) by experts coming from the zonal health department and the regional health bureau. 

It was also discovered that the regular back up technical support by the nearby health centers for the satellite HPs was very much limited and they were also not trusted by the HEWs to obtain their assistance whenever needed. Implanon removal service was not available for the needy clients in the vast majority 202(76.5%) of the HPs. Among 62(23.5 %) of the HEWs who had got Implanon removal services at their HPs for their needy clients, only 37(59.7%) of them had got the service within the last three months, while 23(37.1%) of them had got before three months of the study time. In addition, it was found that 167(63.3%) of the HEWs did not believe their supervisor is available to help them whenever they needed his/her technical assistance. All of these findings call for further exploration of the existing “HC-HP linkages”, which is now praised to be efficient in function and available across board by some government officials.

The study moreover indicated the existence of lack of reference materials, and guidelines for the provision of the overall FP services in general and Implanon insertion in particular. In addition, it revealed that most of the HEWs who had the guidelines and documents did not use it as a reference when serving their clients. One hundred seventy one (64.8%) of the trained HEWs had reported the availability of desktop reference materials for clients’ education on FP methods; however, 70(40.9%) of them did not refer to it when providing Implanon services. A study in Jimma zone also disclosed comparable circumstances that only three service delivery points had a copy of MOH guideline of FP service in Ethiopia (14). However, the study in East Gojjam showed a far better situation in that only 16% of the HEWs were not supplied with reading and reference materials (13). Regarding its utilization, though the materials were available in all except one of the service delivery points of Bahir-Dar zone, most of them were not utilized by service providers (13).

Regarding distance of the HP from the district health office as well as nearby health center; the trained HEWs who were working in the HPs at a distance of less than 10kms from their respective district health offices were found (AOR = 16.82; 95% CI: 4.90- 57.80) times more likely to provide implanon insertion service as compared with those who were working in HPs further away from the district health offices. The study in East Gojjam consistently illustrated that health post distance to the district health office (≥ 10km) were risk factors for a HEW adequate function (11). In the study about the working condition of HEWs in Ethiopia, it was explained as well that in the context of poor transportation and communication systems, distance could have an important impact on logistics, monitoring/supervision, referral, and the overall motivation of the HEWs (12).

This study underscored that the turnover or instability of the HEWs more likely affected the implanon insertion service provision at the HP level. It was disclosed that where at least one of the trained HEWs has left the HP, the trained HEW is less likely to provide implanon insertion service than those who were both working in the same HP after the same training (AOR = 0.16(95% CI; 0.05, 0.48)). Frequent turnover of staff working in the service delivery unit were one of the problems felt by the providers as having negative effect on the quality of their FP service in Jimma zone (14). 

 This study revealed that the participants who had no interest to provide implanon insertion service were found less likely to provide implanon insertion service as compared with those who had an interest towards the service provision (AOR=0.13(95%CI; 0.04,0.41)). Likewise, the odds of current provision of implanon insertion service among the HEWs who had no satisfaction working as a HEW was found to be 0.12 as compared with their counterparts (AOR=0.12;95% CI: 0.04-0.40). In a related study in East Gojjam, future aspiration to stay as HEW was found to be a determinant factor for HEWs functionality; which was explained as the odds of aspiring to stay as HEW (1-3 years), among those nonfunctional health extension workers was 0.98 less than the odds among functional HEWs (11). 

Moreover, the HEWs who had received a regular supervisory visit on Implanon service were (AOR = 2.48 95% CI: 1.08- 5.69) times more likely to provide Implanon insertion service than those who had not received any. A consistent outcome was obtained in the study conducted in East Gojjam that supervision by the health center found to be strongly associated with functionality and described as the odds of being supervised by the health center among non-functional HEWs (11). 

In addition, those trained HEWs who had incorporated their Implanon performance in their last quarter report to their district were found more likely to provide Implanon insertion services (AOR = 11.92; 95% CI: 2.04- 69.52). All of these findings call for further studies to compare with this study findings. 

***Strengths and Limitations of the study*** ***: ***Regarding strength of the study, it is a solitary study that is conducted so far on factors affecting Implanon service provision through trained HEWs in Ethiopia and the study area is one of current government sponsored national scale-up initiatives, of public health importance. Though, being a cross sectional study, it was difficult to establish temporal relationships between variables. Moreover, owing to logistical reasons, the survey was exclusively limited to exploring factors associated to the supply side (physical availability) of the community level Implanon service, but did not attempt to investigate the demand (quality side) factors was a weakness.

## Conclusion

The survey has shown the current status of implanon service provision is not at a promising stage now. The study had also demonstrated a set of determinants that affect “current Implanon insertion by trained HEWs” in Wolaita zone. Among the determinants, distance of health post from district health offices and health center, turnover of trained health extension workers in the health post, interest of trained health extension workers in providing Implanon and their job satisfaction to serve as a health extension workers were the predictors of current provision of Implanon by health extension workers in Wolaita zone. Thus, it will require extra measure to ensure logistics, beyond simply deploying trained providers, if the HEWs driven community level Implanon provision is to materialize. Moreover, improving family planning clients handling at referral facilities will have tremendous impact in sustaining HP level Implanon provision. Likewise, strengthening health center –health post referral linkages is warranted. Moreover, planned supervisory visits by skilled health workers from the nearby HCs/district offices will be recommended. Finally, further studies in similar setting and beyond on the factors affecting in the implanon service provision through HEWs are recommended.
